# Simultaneous Tests of Theaflavin-3,3′-digallate as an Anti-Diabetic Drug in Human Hepatoma G2 Cells and Zebrafish (*Danio rerio*)

**DOI:** 10.3390/nu13124379

**Published:** 2021-12-07

**Authors:** Hui Zhou, Yuanyuan Wu, Eunhye Kim, Haibo Pan, Puming He, Bo Li, Yi Charlie Chen, Youying Tu

**Affiliations:** 1 Department of Tea Science, Zhejiang University, Hangzhou 310058, China; 3150102861@zju.edu.cn (H.Z.); yywu@zju.edu.cn (Y.W.); ehkim@zju.edu.cn (E.K.); pmhe@zju.edu.cn (P.H.); drlib@zju.edu.cn (B.L.); 2 Department of Biosystems Engineering and Food Science, Zhejiang University, Hangzhou 310058, China; apanhaibo@126.com; 3 College of Science, Technology and Mathematics, Alderson Broaddus University, Philippi, WV 26416, USA; chenyc@ab.edu

**Keywords:** theaflavin-3,3′-digallate, anti-diabetic effect, HepG2 cells, diabetic zebrafish model, glucose metabolism enzymes

## Abstract

Theaflavin-3,3′-digallate (TF3) is the most important theaflavin monomer in black tea. TF3 was proved to reduce blood glucose level in mice and rats. However, the elaborate anti-diabetic mechanism was not well elucidated. In this work, human hepatoma G2 (HepG2) cells and zebrafish (*Danio rerio*) were used simultaneously to reveal anti-diabetic effect of TF3. The results showed that TF3 could effectively rise glucose absorption capacity in insulin-resistant HepG2 cells and regulate glucose level in diabetic zebrafish. The hypoglycemic effect was mediated through down-regulating phosphoenolpyruvate carboxykinase and up-regulating glucokinase. More importantly, TF3 could significantly improve β cells regeneration in diabetic zebrafish at low concentrations (5 μg/mL and 10 μg/mL), which meant TF3 had a strong anti-diabetic effect. Obviously, this work provided the potential benefit of TF3 on hypoglycemic effect, regulating glucose metabolism enzymes, and protecting β cells. TF3 might be a promising agent for combating diabetes.

## 1. Introduction

Diabetes mellitus is a worldwide metabolic disease induced by insufficient insulin secretion or insulin resistance and featured by hyperglycemia and metabolism disorder. Chronic hyperglycemia condition causes multiple organ damage and dysfunction which results in high morbidity and mortality of diabetes [1]. This leading killer for human health has become one of the costliest and the most burdensome diseases. Although some drugs such as sulfonylureas, metformin, and acarbose are widely used in clinical treatment of diabetes, the adverse effects such as hypoglycemia, allergic reaction and stomach upsets were also reported [2,3]. Searching for drugs with low cost and no side effects are especially needed.

Lots of dietary flavonoids have been proved to possess anti-diabetic activity due to apparent effectiveness. A double-blinded, randomized and placebo-controlled clinical study showed that dietary supplementation with blueberry bioactive substances could lower fasting plasma glucose and improve insulin sensitivity in diabetic participants [4]. In a cross-sectional study of 1997 females aged 18–76 revealed that high consumption of flavonoid-rich food significantly decreased peripheral insulin resistance [5]. These dietary flavonoids are helpful to alleviate hypercholesterolemia and hyperglycemia. More importantly, these bioactive substances have the advantages of few or no side effects and relatively low costs.

Tea (*Camellia sinensis* (L.) O. Kuntze) is a medicine and food homologous plant. Black tea is a favorite beverage which accounts for approximately 70% of the total tea consumption in the word. Theaflavins (TFs) are key phytochemicals responsible for taste, brisk, color, and antioxidant activity of black tea. TFs are formed from the oxidation of selected pairs of catechins during tea processing. Theaflavin (TF1), theaflavin-3-gallate (TF2A), theaflavin-3′-gallate (TF2B), and theaflavin-3,3′-digallate (TF3) are four major components with relatively high antioxidant capacity [6]. Meanwhile, the aqueous extract of black tea administration was reported to significantly reduce α-glucosidase activity and plasma glucose level in a dose-dependent manner [7]. The regular consumption of black tea could effectively modulate antioxidant capacity and improve the metabolism of glucose [8]. In animal experiments, TFs also showed significant hypoglycemic effect. Drinking TFs caused glucose decline and insulin increase in hyperglycemic rats [9]. Moreover, TF1 administration significantly decreased plasma glucose and altered activities of key enzymes of carbohydrate metabolism, lipid peroxidation markers, antioxidant enzymes in high fat diet and streptozotocin-induced diabetic rats [10]. These results indicated that black tea could be used as a functional food in the dietary therapy for diabetes mellitus.

TF3 has the highest phenolic hydroxyl groups among four major theaflavin monomers, Interestingly, we found that oral administration of TF3-riched complex significantly reduced weight gain and deceased glucose level in obese rat model [11]. Furthermore, group work also demonstrated that TFs improved hepatocellular insulin resistance induced by free fatty acids through promoting mitochondrial biogenesis [12]. It is proved that TFs has complete potency to be developed as an antihyperglycemic agent for combating diabetes mellitus. However, the exact mechanism of TF3 on glucose regulation is still unknown. 

Human hepatoma G2 (HepG2) cell line is obtained from human hepatoma tissue. HepG2 cells are widely used to establish diabetes related models. After glucose induction, HepG2 cells can develop insulin resistance [13] and thus can be used to study the efficacy of drugs in alleviating insulin resistance.

In recent years, zebrafish has emerged as an increasingly disease model and widely applied in the research of blood diseases, diabetes, muscular dystrophy, neurodegenerative disease, angiogenesis and lipid metabolism [14]. Zebrafish has general benefits such as short reproduction cycle, high reproduction frequency, low feeding cost which highlight its application in therapeutic areas and drug discovery [15]. Moreover, it is also verified to be a good model for diabetes research [16] and introduced to evaluate the anti-diabetic function of TF3 monomer. 

In this work, the promoting glucose absorption effect of TF3 was studied in high-glucose-induced insulin resistance HepG2 cells model. Considering that TF3 has a good effect on the characteristics of diabetes, the hypoglycemic effect in alloxan-induced diabetic zebrafish model as well as the cell regeneration promoting function in alloxan-induced β cell damage model were investigated. The specific mechanism of TF3 on regulating glucose metabolism was also elucidated. Moreover, the function of TF3 on combating high-fat and high-glucose induced glucose impairment was systematically discussed.

## 2. Materials and Methods

### 2.1. Materials

TF3 (Figure S1) was separated and purified by a previously established method [17]. The result of liquid chromatography mass spectrometry showed that the purity of TF3 was 92.40% (Figure S2). TF3 was dissolved in distilled water at several concentrations and stored at −20 °C before use.

For cell experiments, Dulbecco’s modified eagle medium (DMEM) and fetal bovine serum (FBS) were purchased from Gibco (Grand Island, NY, USA) and Hyclone (Logan, UT, USA), respectively. Dimethyl sulfoxide (DMSO) and phosphate buffer solution (PBS) was purchased from Sigma (St. Louis, MO, USA). Glucose was purchased from Sinopharm Chemical Reagent Co., Ltd. (Shanghai, China). Reagents were sterilization with 0.22 μm microporous membrane before use. HepG2 cell line was purchased from Institute of Biochemistry and Cell Biology, CAS. (Shanghai, China). 

For zebrafish experiments, alloxan and metformin hydrochloride were purchased from Shanghai Aladdin Bio-Chem Technology Co., Ltd. (Shanghai, China). Glucose was purchased from Hongxing Pharmaceutical (Fuzhou, China). Zebrafish and transgenic β cell fluorescent zebrafish were kindly provided from Hunter Biotech (Hangzhou, China). License number of laboratory animal: syxk (Zhejiang) 2012-0171. Western and IP cell lysates were purchased from Beyotime Biotechnology (Shanghai, China). All other reagents used in this study were of analytical grade and purchased from local reagent supplier, except where stated otherwise.

### 2.2. Cell Culture and Insulin Resistance Model Establishment

HepG2 cells were incubated at 37 °C with 5% CO_2_ in DMEM medium (containing 10% FBS, 100 unit/mL penicillin and 100 μg/mL streptomycin). Cell culture media was changed every 2 days.

After two days’ incubation in 96-well plates, old culture medium was discarded, and PBS was added to wash the cells for three times. Then cells were cultured in DMEM medium with glucose 500 μM for 24 h to obtain insulin resistance model.

### 2.3. Cell Viability Measurement after TF3 Treatment

The viability of HepG2 cells was measured using Cell Counting Kit-8 (CCK-8) kit (7sea Biotech, Shanghai, China). Cells were plated in 96-well plates at a density of 1 × 10^4^ cells per well and treated with different concentrations of TF3 (0, 5, 10, 20, 50, and 100 μM) for 24 h at 37 °C. After treatment, cell culture medium was discarded and 100 μL of 10% CCK-8 solution (diluted in serum-free DMEM medium) was added to each well. After incubation at 37 °C for 1 h, the absorbances of each well were read at 450 nm using a microplate reader (MDC, San Jose, CA, USA).

### 2.4. Glucose Absorption Determination in Insulin Resistance Model Cells

Insulin resistance model cells were divided into several groups. Old culture medium was discarded, and cells were treated with different concentrations of TF3 (1, 5, 10, 20, and 50 μM) or metformin hydrochloride (10 μM) for 24 h. Cell glucose absorption was determined using 2-Deoxy-2-[(7-nitro-2,1,3-benzoxadiazol-4-yl) amino]-D-glucose (2-NBDG) glucose uptake assay kit (Applygen, Beijing, China), according to manufacturer’s instructions.

### 2.5. Zebrafish Culture and Diabetic Model Establishment

The reproduction of zebrafish embryos was carried out after natural mating in pairs. The embryos were cleaned at 6 and 24 h after fertilization then appropriate embryos were selected according to the development stage. After selection, embryos were cultured in specific reverse osmosis water, which contained 200 mg/L instant sea salt. The conductivity of water was 480–510 μS/cm. pH remained 6.9–7.2 and the hardness of water was equal to 53.7–71.6 mg/L CaCO_3_. Because embryos could obtain nutrients from their yolk sac, there was no need to feed within 9 days after fertilization. This feed condition met the standard of Association for Assessment and Accreditation of Laboratory Animal Care (AAALAC).

Zebrafish were randomly divided and transferred into 6-well plates. The density was 30 fish per well. Zebrafish were cultured in water with 1 μM alloxan and 4% glucose for 24 h to obtain diabetic model.

### 2.6. TF3 Toxicity Determination on Zebrafish

Zebrafish were randomly divided into 6 groups (a control group and 5 experimental groups) and each group contained 30 zebrafish. Experimental groups were treated with different concentrations of TF3 (10, 20, 30, 40, and 50 μg/mL) for 24 h, while the control group was treated with water. Eventually, the mortality of each group was calculated.

### 2.7. TF3 Hypoglycemic Effect Determination on Zebrafish Diabetic Model

Zebrafish were randomly divided into 9 groups (a control group, a model group and 7 experimental groups). Diabetic model was established in all groups except control. Experimental groups were treated with different concentrations of TF3 (0.5, 2, 4, 6.7, 10, and 20 μg/mL) or metformin hydrochloride (10 μg/mL). All groups were incubated in an incubator at 28 °C for 24 h. Following this, old culture medium was discarded, and zebrafish were collected into 1.5 mL centrifuge tubes. Each tube contained 5 fish. After drying the water in tubes, 100 μL ethanol was added. Tubes were placed at room temperature for 15 min, transferred to an oven at 60 °C for 2 h. Then 5 μL ultra-pure water was added into each tube. Tubes were again placed at room temperature for 15 min. Finally, 2 μL of solution was taken to measure the glucose content (S) using a blood glucose meter. The glucose absorption reduction effect of TF3 was calculated according to Equation (1):

Hypoglycemic rate (%) = (S_m_ − S_e_)/(S_m_ − S_c_) × 100% (1)

where S_m_ was the glucose content of model group, S_e_ was the glucose content of experimental group and S_c_ was the glucose content of control group.

### 2.8. Western Blot Analysis for Zebrafish Diabetic Model

After incubation at 28 °C for 24 h, old culture medium was discarded, and zebrafish were collected into 1.5 mL centrifuge tubes. Each tube contained 5 fishes. After drying the water in tubes, 150 μL lysis solution (containing PMSF) was added. The zebrafish were ground with a centrifuge tube and then left on ice for 30 min. Then solutions were centrifuged at a speed of 12,000 rpm for 10 min. The supernatant was taken for the following western blot experiment. The concentration of protein was measured using a BCA Protein Assay Kit (Thermo, Waltham, MA, USA).

Western blot analysis was performed as described by Gao [18]. In brief, protein was diluted to a suitable concentration. Then protein was separated by SDS-PAGE gel electrophoresis and transferred to PVDF membranes. After blocked with non-fat milk for 2 h, membranes were incubated at 4 °C overnight in TBST buffer solution and then incubated in another TBST buffer solution. Liquid A and Liquid B of ECL kit was mixed and added to the surface of the membrane. Pictures were obtained using a gel imaging analyzer (Bio-Rad, CA, USA), Image J (National Institutes of Health, Bethesda, MD, USA) was used to analyze the optical density.

### 2.9. Zebrafish β Cell Fluorescence Intensity Determination

Transgenic β cell fluorescent zebrafish were randomly divided and transferred into 6-well plates. β cell injury model was induced by alloxan, according to the method above. Experimental groups were treated with different concentrations of TF3 (2, 6.7, and 20 μg/mL) or metformin hydrochloride (10 μg/mL). After incubation at 28 °C for 24 h, 10 zebrafish from each group were randomly observed and photographed under microscope (Chongqing Photoelectric Instrument Co., Ltd., Chongqing, China). Pictures were analyzed to calculate the fluorescence intensity of zebrafish β cells (S) and evaluate the promoting regeneration effect of TF3 on β cells. The β cell regeneration rate was calculated according to Equation (2):

β cell regeneration rate (%) = (1 − S_e_/S_m_) × 100% (2)

where S_m_ was the fluorescence intensity of model group and S_e_ was the fluorescence intensity of experimental group.

### 2.10. Statistical Analysis

All data were expressed as the mean ± standard deviation (SD) from at least 3 independent experiments, and at least 6 biological replications for each independent experiment have been done. The results were analyzed with Origin 8.0 (OriginLab, Northampton, MA, USA) using the Shapiro–Wilk normality test, one-way analysis of variance (ANOVA) and post-hoc test (2-sided SNK’s test). *p* < 0.05 means there is a significant difference.

## 3. Results

### 3.1. The Effect of TF3 on HepG2 Cells Viability and Morphology

**Figure 1:**
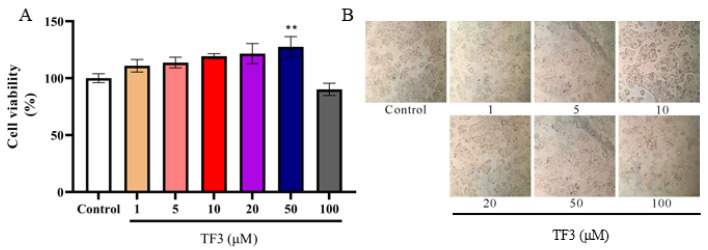
The effect of TF3 on HepG2 cell viability (**A**) and cell morphology (**B**). Control, DMEM medium culture; TF3, DMEM medium containing different concentrations of TF3 (1, 5, 10, 20, 50, 100 μM) treating for 24 h. ** *p* < 0.01 versus control group.

To measure the toxicity of TF3 on HepG2 cells, the viability and morphology of cells treated with TF3 (1–100 μM) for 24 h were investigated. The results showed that TF3 at low concentrations (1–50 μM) had no significant effects on cell viability (Figure 1A). However, when TF3 concentration increased to 100 μM, the viability was decreased to 90%, which suggested that high concentration TF3 might have toxicity on HepG2 cells. Moreover, the morphology observation of HepG2 cell structure was intact, the edge was clear, and no obvious cell damage was found after treated with TF3 at 1–50 μM (Figure 1B). Therefore, the lower and safer concentrations 1–50 μM of TF3 was adopted for the glucose absorption experiments.

### 3.2. TF3 Promotes Glucose Absorption Capacity in Glucose-Induced Insulin Resistance HepG2 Cells

**Figure 2:**
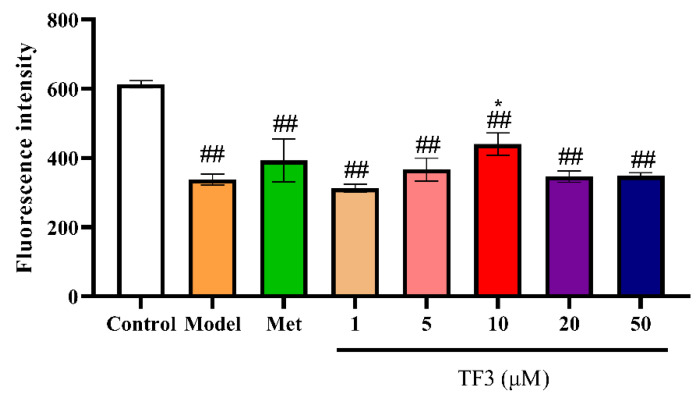
TF3 treatment recovered glucose uptake in glucose-treated insulin resistance HepG2 cells. Control, DMEM medium culture; model, DMEM medium with 500 μM glucose treating for 24 h; TF3, DMEM medium containing different concentrations of TF3 (1, 5, 10, 20, 50 μM) treating for 24 h after glucose-treatment; Met, DMEM medium containing metformin hydrochloride (10 μM) treating for 24 h after glucose-treatment. * *p* < 0.05 versus model group., ## *p* < 0.01 versus control group.

Glucose absorptions in different treatment HepG2 cells were measured by 2-NBDG glucose uptake assay kit (Figure 2). The results showed that glucose induction significantly decreased 2-NBDG absorption capacity in HepG2 cells (44.92%, *p* < 0.01). Glucose metabolism was weakened, and cells were unable to absorb glucose in medium, meaning that insulin resistance cell model was successfully established. With the increasing concentration of TF3 treatment, the glucose absorption capacity of insulin resistance HepG2 cells increased first and then decreased. Cell 2-NBDG uptake significantly increased after 10 μM TF3 treatments (*p* < 0.05). These results suggested that TF3 had the tendency to restore insulin resistance and 10 μM TF3 could efficiently improve the insulin sensitivity of HepG2 cells model.

### 3.3. The Toxicity of TF3 on Zebrafish

Owing to TF3 could promote glucose absorption capacity in cells, the anti-diabetic effect of TF3 in zebrafish was investigated. To access the toxicity of TF3 on zebrafish, the mortalities of zebrafish treated with different concentrations of TF3 (10, 20, 30, 40, and 50 μg/mL) were measured (Figure S3). TF3 was not found toxicity to zebrafish at a concentration lower than 20 μg/mL. However, when TF3 was higher than 20 μg/mL, a dose-dependent toxicity was observed. The IC50 of TF3 was approximately 35 μg/mL. The maximum non-lethal concentration of TF3 (20 μg/mL) was used in the following experiments.

### 3.4. TF3 Reduces Glucose Level in Alloxan-Induced Diabetic Zebrafish

**Figure 3:**
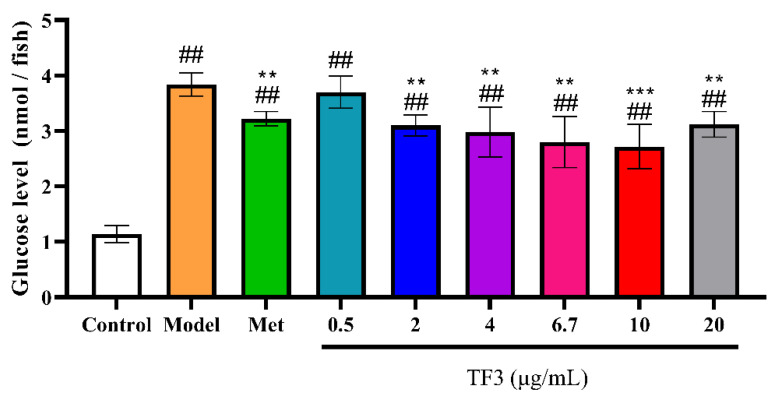
TF3 treatment reduced glucose level in zebrafish. Results were expressed as the mean ± SD (*n* = 5). Control, water culture; model, water with 1 μM alloxan and 4% glucose culture for 24 h; TF3, water with different concentrations of TF3 (0.5, 2, 4, 6.7, 10, and 20 μg/mL) treating for 24 h after alloxan-treatment; Met, water with metformin hydrochloride (10 μg/mL) treating for 24 h after alloxan-treatment. ## *p* < 0.01 versus control group. ** *p* < 0.01 versus model group, *** *p* < 0.001 versus Model group.

To evaluate the hypoglycemic effect of TF3 in alloxan-induced diabetic model, the glucose levels of different treatments were measured (Figure 3). The glucose level in Model group was nearly four-fold of the control group, which meant diabetic model was successfully made. The glucose level decreased by 22.96% in metformin treatment, which suggested metformin had obvious hypoglycemic effect. TF3 2–20 μg/mL could reverse glucose level increase by 27.41–41.48%. Comparing with Met group, TF3 treatment showed a higher hypoglycemic effect at same concentration (10 μg/mL).

### 3.5. TF3 Inhibits Alloxan-Induced Diabetes via Regulating the Expression of PEPCK and GCK

**Figure 4:**
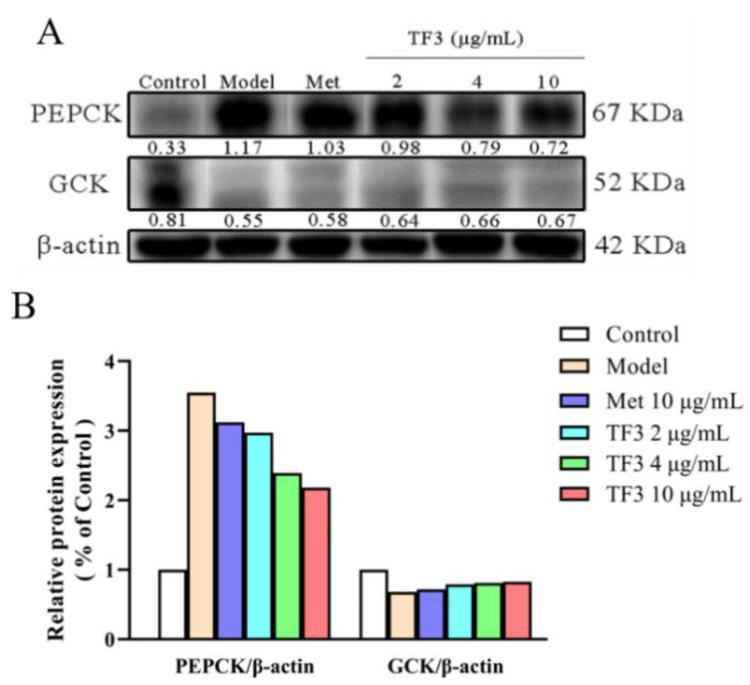
TF3 inhibited alloxan-induced hyperglycemia via regulating PEPCK and GCK expression. (**A**) Blot results and (**B**) relative protein expression. Control, water culture; model, water with 1 μM alloxan and 4% glucose culture for 24 h; TF3, water with different concentrations of TF3 (2, 4, 10 μg/mL) treating for 24 h after alloxan-treatment; Met, water with metformin hydrochloride (10 μg/mL) treating for 24 h after alloxan-treatment.

Phosphoenolpyruvate carboxykinase (PEPCK) and glucokinase (GCK) are two rate-limiting enzymes in glucose metabolism. PEPCK is the key enzyme to control gluconeogenesis. As shown in Figure 4, alloxan increased PEPCK protein level (nearly four-fold of the control group), which meant gluconeogenesis in the model group processed excessively. The treatments of metformin and TF3 could significantly decrease the PEPCK level in diabetic zebrafish. The inhibitory effect of TF3 on PEPCK level showed a dose-dependent manner. TF3 at 2, 4, 10 μg/mL had higher inhibitory effects than metformin. GCK is an isoenzyme of hexokinase, which can phosphorylate intracellular glucose in hepatocyte and prevent the phosphorylated glucose from flowing out, so as to control the blood glucose level. In this work, the GCK protein level drastically reduced after alloxan induction. Different concentrations of TF3 could enhance the expression of GCK while metformin treatment showed little effect. 

In summary, the regulating effects of TF3 on glucose metabolism enzymes of PEPCK and GCK were stronger than those of metformin. These results suggested that TF3 might attenuate gluconeogenesis and improve insulin efficacy through down-regulating PEPCK expression and up-regulating GCK expression.

### 3.6. TF3 Promotes Zebrafish Islet β Cells Regeneration

**Figure 5:**
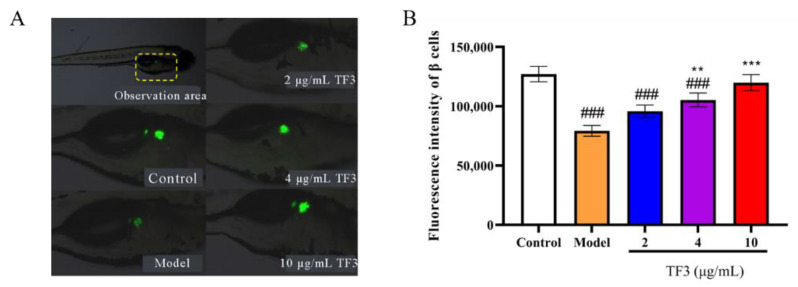
TF3 promoted β cell regeneration from alloxan-induced damage in zebrafish. (**A**) Fluorescence phenotype of zebrafish β cells and (**B**) the fluorescence intensity of β cells. Data were presented as the mean ± SD. Control, water culture; model, water with 1 μM alloxan and 4% glucose culture for 24 h; TF3, water with different concentrations of TF3 (0.5, 2, 4, 6.7, 10, and 20 μg/mL) treating for 24 h after alloxan-treatment. ### *p* < 0.001 versus control group, ** *p* < 0.01 versus model group, *** *p* < 0.001 versus model group.

In this trial, β cell injury model was established to verify the regeneration recovery effect and insulin secretion regulation effect of TF3. The fluorescence intensity in alloxan-treated group was significantly lower than that of control group (*p* < 0.001), meaning that islet β cell disfunction in the model group and β cell injury model was successfully established (Figure 5A). The β cells regeneration rates were 20.80–51.29% with increasing TF3 concentration. TF3 at 4 μg/mL and 10 μg/mL showed significantly differences with model group and the β cells regeneration rate of TF3 at 10 μg/mL showed no significant difference to the control group, (Figure 5B). The results indicated that TF3 could protect β cells from alloxan-induced damage.

## 4. Discussion

Black tea is the largest consumed tea beverage around the world. TFs are a group of benzotropolone derivatives formed from the oxidation of catechins during black tea processing. These important ingredients are beneficial to the quality of black tea [19] and antioxidant capacity [20]. Black tea has been found to effectively improve high-energy diet-induced metabolic syndromes such as hyperlipidemia, obesity, and diabetes in vivo [9,21]. Some researchers also verified the hypoglycemic effect of TFs in vitro such as inhibiting α-glucosidase and regulating glucose level [7,8]. However, the elaborate anti-diabetic mechanism of TF3 monomer was not well reported.

In the present work, TF3 lower than 20 μM had no significant inhibition effects on cell viability, which was consistent with previous results [22]. TF3 was found to significantly recover the glucose absorption capacity at a low concentration (10 μM) in glucose-induced insulin resistance cell model. Up to now, several phenolic compounds have been reported to increase glucose uptake in skeletal muscle cells. Cyanidin-3-glucoside and cyanidin-3-sambubioside had the maximal efficacy on glucose uptake at 10 μM [23], indicating that phenolic compounds have potential anti-diabetic effect. EGCG, the major ingredient in green tea showed the best glucose release reduction effect at the concentration of 25 μM [24]. Here, TF3 monomer showed good effect on improving glucose absorption at a lower concentration, compared to EGCG and mulberry anthocyanin extract in a similar model [25]. These results indicated that TF3 could increase glucose absorption and enhance insulin sensitivity at a relatively low concentration. Given that insulin resistance is an important characteristic of diabetes, next study we focused on the anti-diabetic effect of TF3.

As a new model animal, zebrafish has many advantages such as small size, large sample quantity, short reproduction cycle, high reproduction frequency, low feeding cost, and good repeatability when compared to mice and rats. Hence, it was widely applied in the research of tumor, hematopoietic system disease, cardiovascular disease, etc. [15]. The central nervous system, internal organs, blood, and visual systems of zebrafish are 87% consistent with human’s at molecular and genetic level. Although there are some differences in pancreas morphology between human and zebrafish, the molecular mechanism related to pancreas development and corresponding disease are similar [26,27]. Therefore, a diabetic zebrafish model was well established and applied in the present work to simulate human diabetes. 

In our study, safe range of TF3 (0–20 μg/mL) was selected for the experiments. It turned out that the hypoglycemic rate of TF3 was from 27.41% to 41.48%, which meant TF3 had significant hypoglycemic effects (*p* < 0.01). Moreover, the hypoglycemic effect of TF3 10 μg/mL was stronger than that of 10 μg/mL metformin (*p* < 0.01). Plant extracts or phenolic compounds have already been verified to alleviate alloxan-induced diabetes in zebrafish. For example, *Psychotria malayana* Jack leaf extract and dieckol significantly reduced the blood glucose of diabetic zebrafish to normal level [28,29]. Therefore, including TF3, phenolic compounds have potential effective anti-diabetic effect.

To figure out the anti-diabetic mechanism of TF3 in zebrafish model, western blot analysis of two glucose metabolism enzymes was carried out. GCK is the first rate-limiting enzyme of glucose metabolism in hepatocytes. The synthesis of GCK is regulated by insulin. The deficiency of GCK causes metabolic disorder of glucose in the liver, which is not conducive to the oxidative phosphorylation of glucose and the reduction of peripheral blood glucose, thus causing hyperglycemia, insulin resistance in the liver and the occurrence of type 2 diabetes. It was confirmed that alloxan inhibited the expression of GCK in β cells [30]. PEPCK is another rate-limiting enzyme of glucose metabolism in the lever. PEPCK is regulated by glucose-related hormones. The over-expression of PEPCK results in blood sugar synthesis enhancement and causes imbalance of glucose synthesis and decomposition, which is the main reason of hyperglycemia. PEPCK has been found over-expression in some diabetic models [31,32]. Kim et al. figured out that alloxan and glucose treated group had significantly higher PEPCK level than the control group and dieckol, a polyphenol in *Ecklonia cava*, reversed this change [29]. The glucose level of zebrafish partly depends on the expression of PEPCK. For example, the inhibition of PEPCK expression could cause fasting hypoglycemia in adult akr1a1b^-/-^ mutant zebrafish [33]. In this study, 1 μM alloxan and 4% glucose treatment increased PEPCK level, which promoted the glucose synthesis in liver, resulting in hyperglycemia. In addition, alloxan induced GCK deficiency, which resulted in the lack of glucose oxidative phosphorylation and glucose accumulation. TF3 inhibited the expression of PEPCK and increased the expression of GCK in zebrafish, which regulated the metabolism of glucose.

In zebrafish, alloxan could selectively destroy islets [34] and cause diabetes. Here, we used alloxan and transgenic β cell zebrafish to construct an observable β cell injury model, so as to test the promotion effect of TF3 on zebrafish islet β cells regeneration. The results showed that the fluorescence density of zebrafish β cells increased by 51.29% when treated with 10 μg/mL TF3. The protective effect of TF3 was obviously observed. It has been reported that alloxan or palmitic acid treatment could cause zebrafish β cells damage. After treatment with phenolic compounds, the β cell function could recover. For example, 5-bromoprotocatechualdehyde isolated from *Polysiphonia japonica* could promote β cell proliferation and protect against palmitic acid-induced apoptosis β cell damage [35]. Phenolic compounds from the aerial parts of *Malva verticillate* could recover the reduction of islet tissue caused by alloxan [36]. In this work, TF3 could restore the size of the injured islet, thus promoting the proliferation of islet β cells, increasing insulin secretion in diabetic zebrafish and regulating the blood glucose level. Considering the similarity of zebrafish structure and human body, the results provided theoretical evidence that TF3 inhibited the increase of intracellular glucose level by relieving insulin resistance in human. 

TFs (mainly TF3) was also observed to improve insulin sensitivity in our previous study [11]. Thus, we summarized experimental results of TF3 on anti-diabetic effect in HepG2 cells, SD rats, and zebrafish models (Table 1). In three experimental models, good hypoglycemic effects of TF3 were found when compared with clinical hypoglycemic drug, metformin. These results indicate that TF3 may be a potential anti-diabetic drug for further use.

## 5. Conclusions

In general, TF3 at noncytotoxic concentrations could protect HepG2 cells from glucose-induced insulin resistance by increasing glucose uptake.TF3 could protect zebrafish against alloxan-induced diabetes by regulating blood glucose level. The hypoglycemic effect ofTF3 might be mediated through regulating glucose metabolism enzymes PEPCK and GCK along with promoting islet β cells regeneration. These findings confirmed the anti-diabetic effect of TF3 at the cellular and zebrafish level, and explained the specific mechanism for the first time. TF3 is expected to be developed into a promising agent for combating diabetes.

## Figures and Tables

**Table 1:** Recovery effect of TF3 in different models.

Objects	Model	Treatment	Recovery Rate	Positive Control	Positive Control Group Recovery Rate
HepG2 cells	Insulin resistance model	10 μM TF3	29.31%	Metformin	17.24%
SD rats	Insulin resistance model	100 mg/(Kg·bw) TFs (mainly TF3)	54.93%	/	/
Zebrafish	Diabetic model	2–20 μg/mL TF3	27.41–41.48%	Metformin	18.05%
Transgenic β cell fluorescent zebrafish	β cell injury model	4–10 μg/mL TF3	20.80–51.29%	/	/

## Supplementary Materials

The following are available online at https://www.mdpi.com/article/10.3390/nu13124379/s1, Figure S1: structure of TF3, Figure S2: LC-MS and HPLC-UV profiles of TF3, Figure S3: the effect of TF3 on zebrafish mortality.
