# Supplementary material for: Simultaneous Tests of Theaflavin-3,3′-digallate as an Anti-Diabetic Drug in Human Hepatoma G2 Cells and Zebrafish (Danio rerio)
Source: Nutrients. 2021 Dec 7;13(12):4379. doi: 10.3390/nu13124379 (PMC8704303; doi:10.3390/nu13124379)
Supplement: Supplementary file 1 [file nutrients-13-04379-s001.zip › nutrients-1453753-supplementary.pdf]

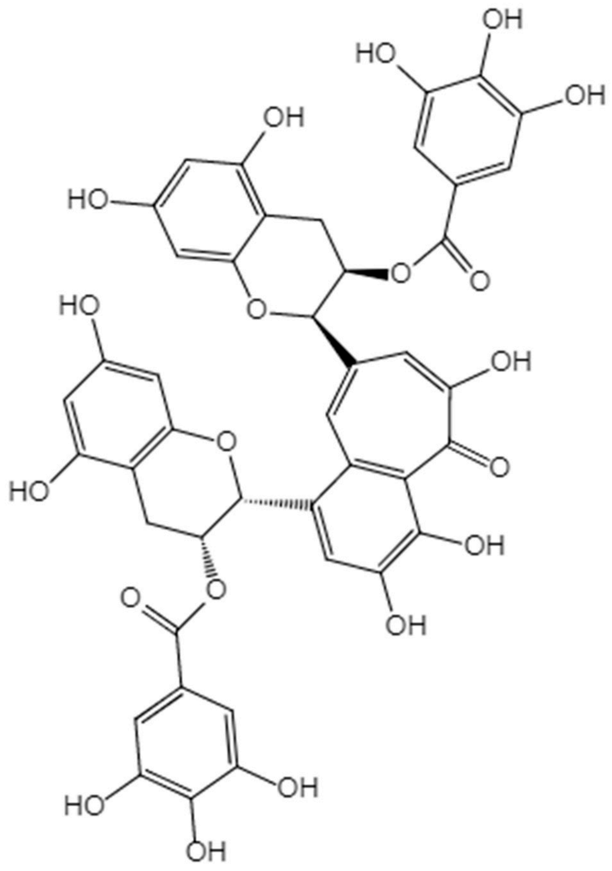

Figure S1. Structure of TF3.

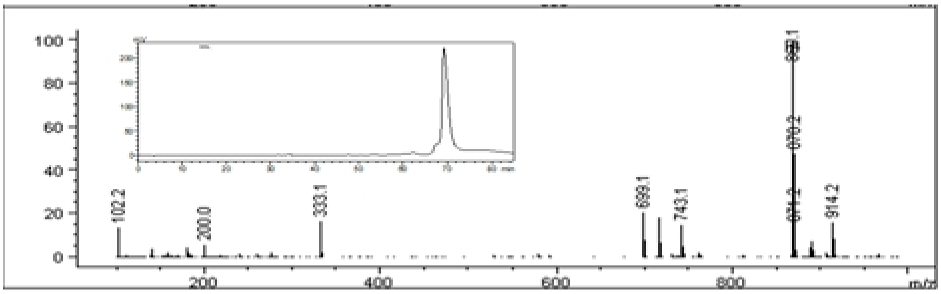

Figure S2. LC-MS and HPLC-UV profiles of TF3

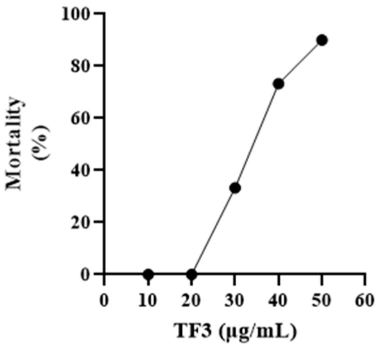

Figure S3. The effect of TF3 on zebrafish mortality.
